# The Role of Dicentric Chromosome Formation and Secondary Centromere Deletion in the Evolution of Myeloid Malignancy

**DOI:** 10.4061/2011/643628

**Published:** 2011-09-27

**Authors:** Ruth N. MacKinnon, Lynda J. Campbell

**Affiliations:** ^1^Victorian Cancer Cytogenetics Service, St Vincent's Hospital (Melbourne) Ltd., P.O. Box 2900, Fitzroy, VIC 3065, Australia; ^2^Department of Medicine (St Vincent's), University of Melbourne, Parkville, VIC 3010, Australia

## Abstract

Dicentric chromosomes have been identified as instigators of the genome instability associated with cancer, but this instability is often resolved by one of a number of different secondary events. These include centromere inactivation, inversion, and intercentromeric deletion. Deletion or excision of one of the centromeres may be a significant occurrence in myeloid malignancy and other malignancies but has not previously been widely recognized, and our reports are the first describing centromere deletion in cancer cells. We review what is known about dicentric chromosomes and the mechanisms by which they can undergo stabilization in both constitutional and cancer genomes. The failure to identify centromere deletion in cancer cells until recently can be partly explained by the standard approaches to routine diagnostic cancer genome analysis, which do not identify centromeres in the context of chromosome organization. This hitherto hidden group of primary dicentric, secondary monocentric chromosomes, together with other unrecognized dicentric chromosomes, points to a greater role for dicentric chromosomes in cancer initiation and progression than is generally acknowledged. We present a model that predicts and explains a significant role for dicentric chromosomes in the formation of unbalanced translocations in malignancy.

## 1. Introduction

Dicentric chromosomes, which have two centromeres, are a well-known feature of cancer cells, and the genome instability and evolution they induce are highly relevant to cancer biology [1, 2]. Although constitutional dicentric chromosomes are much rarer, in those that have been identified there is little evidence of this instability. Our studies have shown that the mechanisms by which dicentric chromosomes are stabilized include the loss of a centromere from a dicentric chromosome making it secondarily monocentric [3, 4], a previously little known mechanism which may be relatively common in cancer evolution. We review evidence that dicentric chromosomes have a greater role in oncogenesis than is currently acknowledged.

During cell division the two centromeres of an unstable dicentric chromosome migrate towards opposite poles at anaphase, causing cycles of breakage and rejoining which create new chromosome arrangements, deletions, and amplifications. This is known as the bridge-fusion-breakage (BFB) cycle [5]. The gene copy number aberrations which are a byproduct of dicentric chromosome instability can create an increased risk of malignancy. Positive selection of these copy number changes can drive clonal evolution [6–10]. 

Copy number aberration (CNA) in the genome—net gain or loss of genetic material—is seen as one of the major causes of cancer. CNA can occur by whole chromosome gain or loss (aneuploidy), simple deletion or duplication of a chromosome segment, or unbalanced translocation where rearrangement between two or more chromosomes produces net gain or loss of material. Isodicentric chromosome formation causes both gain and loss of material, by the joining together of sister chromatids at a double-strand break, and is one method by which unstable dicentric chromosomes can be produced [11, 12].

Dicentric chromosomes in constitutional (nonmalignant) genomes are typically either stable at formation, or have undergone a process of stabilization by mechanisms which have been well studied [13–16]. The processes underlying the stabilization of dicentric chromosomes in cancer cells have not been so well defined. 

We will compare patterns of dicentric chromosome stabilization in the cancer and constitutional settings, including insights gained from our studies of cases of acute myeloid leukemia (AML) and myelodysplastic syndromes (MDS) with unbalanced karyotypes.

## 2. Dicentric Chromosomes in Malignancy

Dicentric chromosomes in cancer are best known as the product of telomere fusion events. Telomeres cap and stabilize the ends of chromosomes. Loss of this protective function, such as by a gradual reduction in the number of DNA telomere-specific repeats as a consequence of repeated genome replication, creates sticky chromosome ends. These can join to each other, with or without complete loss of the residual telomere and subtelomere sequences [17, 18]. Apparent telomere fusion (telomere association) events are characteristic of certain tumours, such as giant cell tumor of the bone [19] and meningioma [20]. Several model systems have been developed for creating and studying dicentric chromosomes *in vitro *or in nonhuman organisms, by artificially induced telomere erosion [7, 10, 18, 21–23]. Recent studies have reported direct molecular evidence for naturally occurring telomere fusion in cancer cells [6, 24].

Studies of dicentric chromosomes *in vivo*, *in vitro*, and in mice with induced telomere dysfunction, support a major role for telomere erosion and subsequent end-to-end chromosome fusion in causing the genome instability which is observed in many types of cancer [6, 8, 21, 22, 24–26], although this has also recently been questioned [27]. The BFB cycle undergone by these dicentric chromosomes is understood to be one of the principle causes of genome instability [11, 26, 28]. 

Unstable dicentric chromosomes can produce rearrangements such as deletion, amplification, inversion, double minute, and ring chromosomes [9, 28, 29]. Micronuclei and other nuclear anomalies can be produced as byproducts of instability. These contain centric or acentric chromosome segments which have not segregated to either daughter cell [26, 30]. Lagging of dicentric chromosomes so that they are then lost to a micronucleus, or missegregation of the whole dicentric chromosome to one daughter cell, can produce whole chromosome aneuploidy [21, 26]. 

Studies of transformed cell lines and premalignant cells have shown that with continued cell division and shortening of the telomeres, the cells enter a period of crisis, which is associated with end-to-end fusion of the chromosomes and genome instability, causing genome aberration. These cells with aberrant genomes would normally undergo senescence, but can survive if p53-induced apoptosis is inactivated. Telomere length and stability are restored by upregulation of telomerase or the alternative lengthening of telomere (ALT) mechanism [6, 8, 31–34]. 

**Figure 1:**
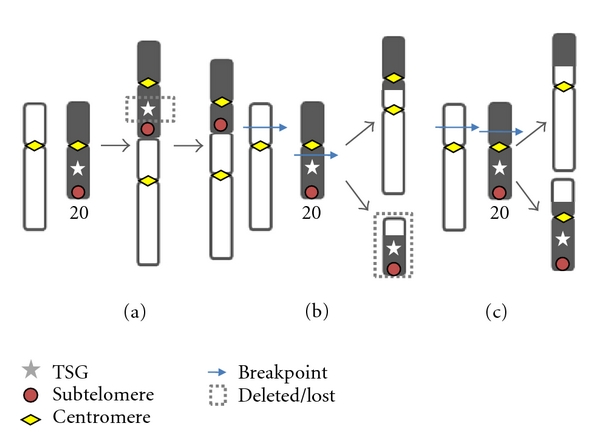
Translocations described in the text. (a) *Telomere Fusion*, creating a dicentric chromosome with minimal DNA loss but potentially causing secondary copy number aberration via the BFB cycle. (b) *Dicentric Translocation*, a reciprocal translocation producing a dicentric chromosome, and an acentric chromosome which is lost at cell division; (c) *Balanced Reciprocal Translocation*, producing two monocentric chromosomes—another event is required for CNA to occur. Our model proposes that (a) and (b), which both produce a dicentric chromosome, are the major causes of unbalanced translocation.

Translocation between two or more chromosomes with interstitial breakpoints (reciprocal translocation) can also produce dicentric chromosomes (Figure 1). Hematological malignancies have the most well characterized chromosome abnormalities of any malignancy, and most dicentric chromosomes reported in hematological malignancies have a morphology consistent with this type of rearrangement rather than telomere fusion. Morphology can be misleading however, and there is now evidence that over half of the dicentric chromosomes involving chromosome 20 in myeloid malignancy are formed by telomere fusion events [35]. It is not yet known whether this pattern extends to other chromosomes.

Dicentric chromosomes which have been identified in hematological malignancies include the recurrent dic(17;20) [36, 37] and dic(5;17) [38] in MDS and AML and the dic(9;20) in acute lymphoblastic leukemia [39], as well as a range of other abnormalities [3, 39–41]. These are usually interpreted as unbalanced reciprocal translocations (see Figure 1), with breakpoints often described at or near the centromere [40, 42, 43]. The dic(9;20) is an unusual case. While most dicentric chromosomes, including the recurrent dic(17;20) [4], appear to have a range of breakpoints on both chromosomes, the dic(9;20) has been shown to have breakpoints within a single gene on 9p, *PAX5*, creating a fusion gene between this gene and a number of different genes on 20q [44–47]. 

Studies which focus on the identification of dicentric chromosomes in hematological malignancies have tended to uncover a higher incidence of dicentric chromosomes than is usually reported. In one of the rare studies into the incidence of dicentric chromosomes in myeloid malignancy, Andersen and Pedersen-Bjergaard [42] identified a dicentric chromosome in 15% (27/180) of consecutive cases of therapy-related AML (t-AML) and t-MDS and a much lower incidence in *de novo *disease (0.4%). The combined incidence for AML and MDS was 8%. Callet-Bauchu et al. [41] identified a high incidence of dicentric chromosomes (10/14) among 17p translocations in chronic B-lymphoid disorders.

## 3. Dicentric Chromosomes in Myeloid Malignancy

A diverse range of cytogenetic aberrations has been described in the myeloid malignancies AML and MDS, including highly specific balanced translocations producing fusion genes, and copy number aberrations. Our studies of *in vivo* karyotype abnormalities in patients with these diseases have revealed a high incidence of dicentric chromosomes in unbalanced translocations involving chromosome 20. 

We characterized the centromere and chromosome content and organization of 32 unbalanced chromosome 20 translocations, including thirteen unbalanced 17;20 translocations [3, 4, 36, 48]. These 32 cases had been identified as having apparent monosomy 20 (24 cases) and/or a 17;20 translocation (13 cases) (five cases fulfilled both criteria). Most had lost the putative tumor suppressor gene (TSG) region at 20q12. 

**Figure 2:**
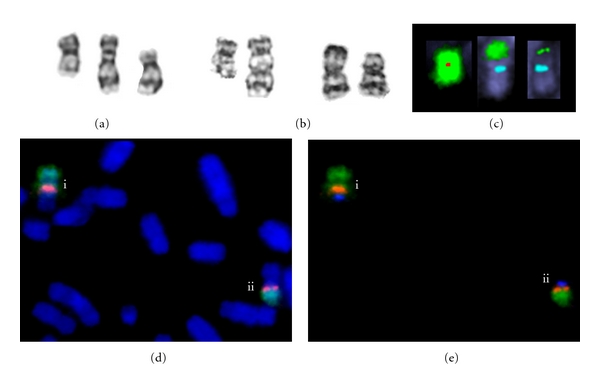
(a-b) G-banded chromosomes. (a) A typical stable dic(17;20) (centre) with the normal 20 (left) and 17 (right) from the same metaphase (Case  3 of Patsouris et al. [36]). (b–d) examples from a case with a highly variable dic(17;20) (Case  6 of MacKinnon et al. [4], SVH01 of MacKinnon et al. [48]). (b) Left, normal chromosome 20 and unstable dic(17;20) from a single metaphase. Right, two secondarily monocentric chromosomes with a 17 centromere (der(17)) from the same patient—each was derived from the primary dic(17;20) translocation by rearrangements which included loss of the 20 centromere. Both show a monocentric morphology and neither is easily recognizable as a pseudodicentric chromosome. (c) FISH images of the normal chromosome 20 and the two der(17)s illustrated in (b) (from a single metaphase). False colour images after FISH show the 20 centromere (red) (missing from the der(17)s), 17 centromere (aqua) (present in the der(17)s), and chromosome 20 content (green). (d-e) Two dic(17;20)s from the case illustrated in (b) and (c), both of which have secondary rearrangements bringing the centromeres closer together. (d) Blue, chromosomes stained with DAPI; green, whole chromosome 20 paint; red 20 centromere; (e) Blue, 17 centromere; green, whole chromosome 20 paint; red, 20 centromere. The derivatives shown have (i) inversion of chromosome 20 material and (ii) deletion of intercentromeric material.

Eight cases of dic(17;20) had a typical morphology comprised of 20p, 17q, and the proximal regions of 20q and 17p between the two centromeric constrictions [3, 36] (Figure 2(a)). A further five cases had a variant 17;20 translocation derived by secondary rearrangement of a primary dicentric chromosome [4, 48] (e.g., Figures 2(b)–2(e)). Thus, every one of thirteen 17;20 translocation products that we identified had been formed as a dicentric chromosome [4, 36, 48],

Twenty-one of 24 cases (87.5%) with apparent monosomy 20 (including the five variant 17;20 translocations) were shown to have a primary dicentric translocation, and only six of these appeared to have no secondary rearrangement or epigenetic inactivation of a centromere (Table 1) [3]. There was not enough information to determine whether the translocations of the remaining three cases had been derived from dicentric chromosomes.

These findings point to a hidden body of dicentric chromosomes in myeloid malignancy, that can only be identified by detailed characterization. In a review of dicentric chromosomes in hematological malignancies, Berger and Busson-Le Coniat [40] reported that the identification of dicentric chromosomes is increased when fluorescence *in situ *hybridization (FISH) with specific *α*-satellite DNA probes is used and suggested that the true incidence is higher than realized. Our studies show that even the use of centromere probes will not identify all dicentric translocations, because some primary dicentric chromosomes become secondarily monocentric. Our evidence points to a frequency of primary dicentric chromosomes in AML/MDS which is higher than the 8% reported by Andersen and Pedersen-Bjergaard [42] and is potentially as high as the frequency of unbalanced translocations in AML/MDS. In our laboratory during 2009 and 2010, the frequency of karyotypes with unbalanced translocation in new cases of MDS or AML was 18%.

## 4. Stabilization of Dicentric Chromosomes in Myeloid Malignancy

These detailed studies of dicentric chromosomes in cases of AML and MDS gave us the opportunity to identify some of the mechanisms by which dicentric chromosomes had been stabilized. In 18/29 primary dicentric chromosomes, some or all of the cells had undergone secondary events which could be interpreted as producing a more stable derivative. A summary of these different secondary events is presented in Table 1. One case had a highly unstable dic(17;20) with 26 Mb between the centromeres, that had produced a wide range of derivatives (Figure 2) [4]. The variety of rearrangements is reminiscent of those described by Riboni et al. [2] in a dicentric chromosome which was produced by telomere fusion *in vitro*.

Centromere suppression occurred by functional inactivation (which was assumed when *α*-satellite DNA was still present but there was no centromeric constriction) or by excision of the *α*-satellite DNA. (Centromere suppression refers to loss of centromere function, regardless of how this is achieved). Loss of centromere-specific *α*-satellite DNA from the chromosome was the most common type of secondary rearrangement. The dicentric chromosome of 11 cases had become secondarily monocentric, by deletion of a centromere from a primary dicentric chromosome. In some cases the excised segment containing the centromere was retained in the cell as what appeared to be a ring chromosome (Table 1). 

**Figure 3:**
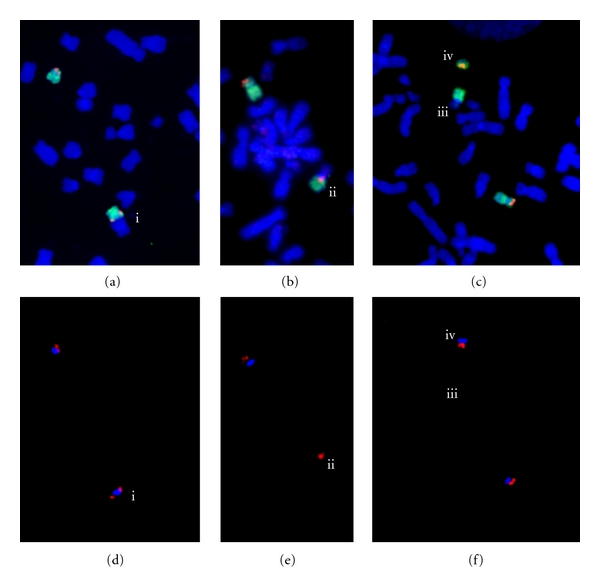
FISH images of three partial metaphases from a case with a dic(16;17) and derivatives formed by loss of the 17 centromere. The normal 17 is also present in each image. (a–c) Blue, chromosomes; red, *TP53* (a 17p13 locus); green, whole chromosome 17 paint; (d–f). Each image shows the same view as the image above it but the labelled 17 centromere is visible: blue, 17 centromere; red *TP53*. (a) (i) a dic(16;17)(q12.1;p13). (b-c) derivatives of the dic(16;17). (b) (ii) a der(16) (the 17 centromere has been lost). (c) (iii) a der(16) and (iv) the deleted segment containing the 17 centromere and *TP53*. Methods for FISH were as described in MacKinnon et al. [4].

We identified loss of the 20, 6, or 17 centromere in these cases [3, 4]. We have also identified cases where there has been loss of the 17 centromere from a primary dicentric chromosome not involving chromosome 20 (RNM, unpublished results; Figure 3). Similar studies will be needed to determine if centromere deletion occurs more widely in dicentrics involving other chromosomes and in other malignancies.

Some secondary rearrangements were produced by intercentromeric deletion or inversion of the inferred primary dicentric chromosome (Figures 2(d), 2(e)). These rearrangements produced dicentric derivatives with a greatly reduced intercentromeric distance, apparently resulting in stable dicentric chromosomes. The stable dicentric chromosomes showing no evidence of secondary events had short intercentromeric distances, supporting studies in constitutional dicentric chromosomes which show that this property renders the dicentric chromosome stable [14, 49]. 

More than half of the dic(20;var) which had lost the 20q12 TSG region had done so by an interstitial deletion, retaining the distal, subtelomeric 20q region [35]. Based on evidence that dicentric chromosomes can occur by telomere fusion, the simplest explanation of our findings was that an unstable primary dicentric chromosome had been formed by telomere fusion between chromosome 20 and another chromosome, followed by positive selection of a derivative with a secondary 20q12 deletion (see Figure 1). However, the alternative explanation, translocation between a preexisting del(20q) and another chromosome, could not be discounted. 

We have described five cases of dic(17;20) with interstitial deletion of 20q suggestive of telomere fusion and subsequent deletion of the 20q12 TSG containing region [4, 35, 48]. A telomere fusion between 17p and 20q would produce a dicentric chromosome with an intercentromeric distance of 56 Mb. According to studies of constitutional dicentric chromosomes [14, 49] a dicentric chromosome with this intercentromeric distance would be unstable. Thus, the secondary deletion of 20q12 could fulfill two roles: loss of a TSG and stabilization of the dicentric chromosome.

We have also reported localized amplification of a section of 20q (20q11.21), in both MDS and AML, the development of which was probably aided by its position between the centromeres of unstable dicentric chromosomes [3, 48]. We have suggested that this selectively amplified region contains an oncogene [3, 48]. As well as deletion, the BFB cycle can cause amplification of material between the centromeres of a dicentric chromosome. Positive selective pressure would tend to favor amplification of an oncogene in this position. Gain of the whole abnormal chromosome in other cases, rather than localized amplification, provides further support for on oncogenic role for a gene in this region. Further instances of amplification of 20q11.21 have been identified since these publications (RNM, unpublished observations).

## 5. Centromere Deletion

Centromere deletion or excision has only rarely been described previously. Nevertheless, it was a significant event in our series (occurring in 11 of 29 primary dicentrics) and was more frequent than functional inactivation. FISH with BAC (bacterial artificial chromosome) clones was used to define the breakpoints of 20 centromere deletion [48], which fell into two main categories. Some had deletion breakpoints close to the centromere, retaining part or all of the flanking BACs (two published cases [48] and one unpublished case (RNM, unpublished results)), while others had excision of a broader section including 20q11.21, and the excised fragment was retained in the cell as either a ring or marker chromosome (4 cases). We have also observed larger deletions spanning the centromere without retention of the deleted segment (chromosome 17, one case and chromosome 20, two cases) [4, 48]. Some cases had multiple clones with different centromere deletion events. There was no FISH evidence of partial retention of the centromere in any of these cases.

In the 1970s it was suggested that centromere suppression could be a result of either deletion of the centromere or functional inactivation [50–52]. However, molecular studies since then have identified epigenetic mechanisms for suppressing centromeres, that is, loss of the centromere proteins which form the kinetochore, produces a functionally inactive centromere [13, 53]. These studies were carried out on constitutional dicentric chromosomes, but it seems to have been generally assumed that functional inactivation is also the mechanism of centromere suppression in cancer cells (e.g., [40]). Although dicentric chromosomes are commonly found in cancer cells, mechanisms stabilizing these dicentric chromosomes have not been well studied. 

In humans, centromere deletion from a dicentric chromosome has been noted most often in constitutional dicentric Y chromosomes [54–57], but also in a handful of other cases [58, 59]. The *α*-satellite DNA was found to be only partially removed in some of these, including the two examples of isodicentric Y chromosome which were studied in detail by Tyler-Smith et al. [54], a landmark study which used these deletions to identify *α*-satellite DNA as the sequence marking the centromere. In some cases [55, 56, 58, 59] the excised section, including the centromere, was preserved in a small stably transmitted marker chromosome. Rivera et al. [55, 56] identified at least six cases with pseudodicentric Y chromosomes in which the excised Y centromere was still present, in a small marker chromosome. These authors have suggested that centromere excision from pseudodicentric Y chromosomes with retention of the excised section is under-diagnosed, because these marker chromosomes can usually only be detected by FISH [55]. 

Centromere deletion has also been identified in yeast. Artificially created dicentric chromosomes in *Saccharomyces cerevisiae *can be stabilized by deleting a section of DNA containing one centromere [60, 61]. 

Stimpson et al. [7] observed partial *α*-satellite deletion in dicentric human chromosomes that were created *in vitro*, in fibrosarcoma cell lines. However, these dicentric chromosomes had been artificially induced by *in vitro *abrogation of telomere function.

Our studies identifying centromere deletion in MDS and AML combined FISH for specific centromeres with traditional cytogenetics, multicolor FISH (M-FISH), and multicolor banding (M-BAND) [3, 4, 48]. To our knowledge ours are the first studies identifying centromere deletion from naturally occurring cancer chromosomes. Centromere deletion or excision was the most common mechanism of dicentric chromosome stabilization that we identified in abnormalities of chromosome 20. This suggests that there is a significant rate of unidentified centromere deletion in cancer genomes.

## 6. Functional Inactivation of a Centromere

While dicentric chromosomes are relatively common in cancer cells, they are rare in the constitutional setting, probably because most events causing genome imbalance are incompatible with embryo viability. Nevertheless, most studies of *in vivo* dicentric chromosome stabilization in humans have been carried out on constitutional dicentric chromosomes discovered through clinical cytogenetic analysis. Stabilization of these dicentric chromosomes has generally occurred by the time of discovery [13–16].

The most commonly reported constitutional dicentric chromosomes are Robertsonian translocations and isodicentric X chromosomes—which have lost nonessential material. Stability is typically achieved through close physical proximity of the centromeres [14, 49] or suppression of one of the centromeres [16, 62, 63].

In a series of isodicentric X chromosomes in patients with Turner syndrome, Sullivan and Willard [49] determined that an intercentromeric distance of 12 Mb or less is compatible with a stable, functionally dicentric chromosome (in which both centromeres are active), whereas a larger intercentromeric distance is not compatible with stability. Dicentrics with more material between the centromeres (at least 34 Mb in their examples and 15 Mb in a recent paper by Ewers et al. [64]) consistently had only one active centromere. Available evidence points to a requirement for rapid stabilization of a dicentric chromosome with well-separated centromeres, if the (nonmalignant) cells are to remain viable [49, 65, 66]. 

Centromere suppression in constitutional dicentric chromosomes with well-separated centromeres has most often been shown to be achieved by functional inactivation, producing a pseudodicentric chromosome. Functional inactivation occurs by loss of the centromere proteins which assemble at the kinetochore and define an active centromere [13, 53, 67–70]. Antibodies to some of these epigenetic markers, CENP-A, CENP-C, and CENP-E, have commonly been used to distinguish between functionally active and inactive centromeres [7, 13, 53]. Page and Shaffer [14] showed that the centromere-specific alpha satellite DNA of both centromeres was maintained in dicentrics with one inactive centromere.

Even in stable dicentric chromosomes with closely apposed centromeres, there is usually a mixture of cells with one or two active centromeres, as has been shown in Robertsonian translocations [13, 14], isodicentric X chromosomes [49, 71], and other constitutional dicentrics [50, 66]. The patterns of inactivation seen in parent-child pairs were consistent with stable transmission of the centromere in either the active or inactive state, with a gradual progression towards loss of functionality of either centromere if both were inherited in the active state [14]. In cultured cells there tended to be a higher proportion of functional monocentrics (structural dicentrics with one active centromere) [13, 14], consistent with a tendency to centromere inactivation but not reactivation. 

It is not known whether centromere reactivation occurs *in vivo* in humans. The presence of CENP-A is usually a prerequisite to kinetochore assembly in humans [68, 72–74]. However, centromere DNA lacking centromere proteins can be reconstituted (reactivated) in yeast, maize, and mammalian artificial chromosomes *in vitro* [75–78]. Also, in rare instances a new human centromere (neocentromere) can be created at a position naïve of centromere *α*-satellite DNA by assembly of the necessary proteins, to preserve a marker chromosome that lacks a native centromere [79]. This suggests that reactivation of a human centromere *in vivo* is possible, but there have been no conclusive reports of this occurring [14, 15, 59, 80, 81]. 

Stimpson et al. [7] looked at mechanisms of stabilization of artificially created dicentric chromosomes. The fate of the centromeres in these dicentric chromosomes was followed from the time the dicentric chromosomes were formed. There was progressive centromere suppression after several generations by functional inactivation and, less commonly, partial deletion of *α*-satellite DNA at the centromeres as noted above. 

Stabilization of double-strand DNA breaks in dicentric chromosomes by the addition of new telomeres has been described in mouse embryonic stem cells and tumor cells [17, 82, 83]. Similarly, splitting of a dicentric chromosome into two monocentric derivatives has been described in humans [2] and yeast [84].

## 7. Identification of Dicentric Chromosomes and Secondary Rearrangements

A number of factors can explain why secondary centromere deletion in malignancy had not been recognized earlier. These include the tendency to explain pseudodicentric chromosomes as having been derived by centromere inactivation and the infrequency of testing for the presence of centromeres in routine metaphase cytogenetic analysis. In our experience, monocentric chromosomes derived from dicentric chromosomes were often only identified after a detailed analysis of centromere content, chromosome content, and organization. 

FISH studies to detect pairs of centromeres are not usually carried out on metaphase chromosomes during diagnostic cancer cytogenetic analysis. The chromosomes that are most likely to be recognized as dicentric are those stable primary dicentric chromosomes that have two distinct centromeric constrictions or a recognizable pseudodicentric morphology spanning both centromeres. Other secondary monocentric chromosomes are morphologically unrecognizable because of the altered morphology around the centromere, as is the case with chromosome 20 morphology after the centromere has been deleted [3, 85] (Figure 2(b)). Furthermore, dicentric chromosomes that break into two monocentric derivatives might not be identified as having been derived from a primary dicentric chromosome. 

The use of array karyotyping (single nucleotide polymorphism—SNP—and comparative genomic hybridization—CGH—arrays) on its own for cytogenetic analysis will not identify dicentric chromosomes, telomere fusion, or centromere deletion. Centromeres and telomeres are not represented on standard microarrays, nor do microarrays give information on translocation partners or chromosome organization. As residual telomere sequence can be retained at the site of telomere fusion [2, 6, 86], there will not necessarily be loss of the distal part of the chromosome that is detectable by microarrays, in dicentric chromosomes formed by telomere fusion. In the two cases of apparent telomere fusion that we analyzed by array CGH [48], there was no detectable terminal deletion of 20qter, nor did the arrays show any indication of the centromere loss that had occurred. This may have clinical implications. Dicentric chromosomes may have a poorer prognosis because they can be subject to more rapid selective evolution.

If on any derivative chromosome both translocation partners contain material spanning the centromeres, this is the strongest indication that there was a primary dicentric translocation. A method for identifying chromosome content (array karyotyping, M-BAND, or traditional banding) in conjunction with FISH for the centromeres (using a pancentromere probe or specific centromere probes or, more efficiently, multicolor centromere FISH (cenM-FISH) which will identify all the centromeres in one step [87]) and a method for identifying translocation partners (M-FISH or traditional banding) can therefore help identify dicentric chromosomes, including primary dicentric chromosomes which have lost one of the centromeres. M-BAND [88] gives information on gross chromosome content and organization within an individual cell and also helps define different clones. It can help shed light on the rearrangements that have occurred, if this information is not provided by standard karyotyping. It may not be practical to carry out this level of analysis in routine diagnostics, but for a full understanding of the causes and consequences of unbalanced translocation in a research context, it may be essential.

## 8. The Tip of the Iceberg?

Our studies highlight a higher incidence of primary dicentric chromosome formation in unbalanced chromosome 20 abnormalities than has previously been recognized. The dic(20;var) appears to be an example of an unstable dicentric chromosome creating selectable derivatives that can allow rapid clonal evolution.

Our studies suggested a greater role for telomere fusion in creating dicentric chromosomes in myeloid malignancy than has been previously acknowledged. Environmental and biological factors associated with telomere erosion, that is, advancing age and chemical exposure, are also risk factors for developing MDS and AML [43, 89–91], and so identifying a high incidence of telomere fusion as an oncogenic event may help identify the causes of these diseases. Complex karyotypes often include many unbalanced translocations, frequently of unknown composition, and the resulting chromosomes are typically assumed to be monocentric unless there is clear evidence to the contrary. Given the apparently high incidence of unidentified chromosome 20 dicentrics in myeloid malignancy, we suggest that other unbalanced translocations in AML, MDS, and other malignancies may also harbor a higher incidence of dicentric chromosomes than is currently recognized. This would suggest that the instability associated with dicentric chromosomes is a much more significant factor in cancer than is apparent. We propose a model that both explains the high incidence of dicentric chromosomes in unbalanced chromosome 20 translocations and makes the more general prediction that dicentric chromosome formation is a major mechanism for unbalanced chromosome translocation in malignancy.

## 9. Model for the Formation of Unbalanced Translocations by Dicentric Translocation

This model suggests that most unbalanced translocations are formed by one of two mechanisms producing a dicentric chromosome, *dicentric translocation*, and *telomere fusion*. Genome imbalance is a direct consequence of both of these events. Three types of translocation are defined.

- *Telomere Fusion* (end-to-end joining of chromosomes at the telomeres): this is a balanced rearrangement (apart from possible telomere and subtelomere loss), but if the centromeres are well separated, the chromosome is unstable and further rearrangements are likely—including oncogenic deletions and amplifications—creating an unbalanced translocation product.
- *Dicentric Translocation*: a simple reciprocal translocation producing a dicentric and an acentric chromosome. As the acentric chromosome is lost during mitosis, this becomes an unbalanced translocation.
- *Balanced Reciprocal Translocation*: a simple reciprocal translocation producing two monocentric chromosomes. There is no net gain or loss of material and another event would be required to produce copy number aberration. Therefore we suggest that this is not a usual cause of genome imbalance, contrary to the tacit assumption that the products of most oncogenic unbalanced translocations are monocentric.

The novel aspect of this model is that it predicts that dicentric chromosomes play a much greater role in oncogenesis than is currently appreciated and gives a smaller role to balanced reciprocal translocation in the generation of copy number imbalance. A significant role for dicentric chromosomes produced by telomere attrition in causing genome instability is already recognized. Our chromosome 20 studies provide evidence for both mechanisms of dicentric chromosome formation and support only a minor or coincidental role for reciprocal translocation producing two monocentric chromosomes.

## 10. Conclusions

Dicentric chromosomes are rarely identified in constitutional genetics. However, in cancer cells the formation of dicentric chromosomes is a well-recognized event, which may contribute to the malignant phenotype and clonal evolution. We have explored the patterns of stabilization of dicentric chromosomes in constitutional karyotypes, cancer cells, and cells in tissue culture.

Our studies have shed a light on the role of dicentric chromosome formation in myeloid malignancy. Many primary dicentric chromosomes escape detection when traditional or molecular karyotyping is used to characterize genome aberrations. We have uncovered a significant role for centromere deletion or excision in the evolution of myeloid malignancy, which raises the possibility that this occurs more widely, and in other cancers. More detailed studies combining molecular karyotyping (which allows precise breakpoint definition and genome-wide detection of copy number aberration) with FISH studies (which allow chromosome organization and centromere content to be determined) will lead to a better understanding of the role of dicentric translocations in cancer.

##  Conflict of Interests

The authors declare that there is no conflict of interests.

## Figures and Tables

**Table 1:** Patterns of secondary chromosome aberration derived from 32 primary dicentric (20;var) chromosomes in cases of AML and MDS described in previous publications. In total eleven cases had some mechanism of secondary centromere deletion and had retained or lost the deleted segment, and eight had another type of rearrangement which produced an altered (secondary) dicentric chromosome which retained both chromosomes (four cases had mixtures of clones exhibiting both mechanisms).

Secondary event	MacKinnon and Campbell [3]	MacKinnon et al. [4]	Patsouris et al. [36]	MacKinnon et al. [48]	Total
No change	6	4	1		11
Centromere inactivation	3				3
Intercentromeric deletion	1				1
Inversion reducing intercentromeric distance	3				3

*Cases with secondary monocentric chromosomes*:					
Mixture of clones: intercentromeric deletion/centromere deleted		1			1
Mixture of clones: intercentromeric deletion/centromere excised and retained in a ring chromosome	1				1
Centromere deleted	5				5
Centromere excised and retained in a ring chromosome	2				2
Complex mixture of clones: dicentric and secondary rearrangements (centromere deletion, excision of centric ring, inversion, deletion)		1		1	2

Total	21	6	1	1	29
